# Identities and recurrence relations of special numbers and polynomials of higher order by analysis of their generating functions

**DOI:** 10.1186/s13660-018-1815-7

**Published:** 2018-08-22

**Authors:** Yilmaz Simsek, Daeyeoul Kim

**Affiliations:** 1 Department of Mathematics, Faculty of Science, Akdeniz University, Antalya, Turkey; 2 Department of Mathematics and Institute of Pure and Applied Mathematics, Chonbuk National University, Jeonju-si, South Korea

**Keywords:** 11B68, 05A15, 05A19, 26C05, 30C15, Apostol–Bernoulli numbers and polynomials, Bernoulli numbers and polynomials, Special numbers and polynomials, Generating functions

## Abstract

The aim of this is to give generating functions for new families of special numbers and polynomials of higher order. By using these generating functions and their functional equations, we derive identities and relations for these numbers and polynomials. Relations between these new families of special numbers and polynomials and Bernoulli numbers and polynomials are given. Finally, recurrence relations and derivative formulas, which are related to these numbers and polynomials, are given.

## Introduction

Many special numbers and polynomials have been found by researchers in recent years. These numbers and polynomials have various applications in mathematics and related areas. It is well known that special polynomials have been applied to model including real phenomena (*cf*. [1–15]; see also the references cited therein). In this work, we define a new family of special numbers and polynomials of higher order with their generating functions. Therefore, motivation of this paper is to derive some identities and recurrence relations for these new families of special numbers and polynomials of higher order by using generating functions, functional equations, and partial derivative formulas.

In order to derive the results of this paper, we need generating functions for new families of special numbers and polynomials.

Kim et al. [3, Eq. (2.5)] defined the following generating function for the special numbers $\mathfrak{s}_{n} ( a;q ) $:

1 $$ F ( t;a,q ) = \biggl( \frac{t\log a}{\log q}+1 \biggr) \frac {q ( q-1 ) }{qa^{t}-1}=\sum _{n=0}^{\infty}\mathfrak{s}_{n} ( a;q ) \frac{t^{n}}{n!}. $$

By help of the above generating function, Kim et al. [3, Eq. (2.5)] also defined the following generating function for the special polynomials $\mathfrak{s}_{n} ( x;a;q ) $:

2 $$ \begin{aligned} &G ( t,x;a,b,q ) = a^{tx}F ( t;a,q ) , \\ &G ( t,x;a,b,q ) = \sum_{n=0}^{\infty} \mathfrak{s}_{n} ( x;a;q ) \frac{t^{n}}{n!}. \end{aligned} $$

We summarize the content of this paper as follows:

In Sect. 2, relations between a new family of specials numbers and polynomials, Apostol–Bernoulli numbers and polynomials, and also Bernoulli numbers and polynomials are given.

In Sect. 3, generating functions for new families of special numbers and polynomials of higher order are given. By using these functions with their functional equations, many formulas, identities, and relations for these numbers and polynomials are given.

In Sect. 4, by using partial derivative formulas for generating functions of new families of special numbers and polynomials of higher order, recurrence relations for these numbers and polynomials are given. Finally, we complete this paper with conclusion section.

## Relations between Bernoulli type numbers and polynomials

We give relations between Apostol–Bernoulli numbers and polynomials and also Bernoulli numbers and polynomials.

Apostol Bernoulli polynomials of order *k* are defined by the following generating function:

3 $$ F_{AB} ( t;x,\theta ) = \biggl( \frac{t}{\theta e^{t}-1} \biggr)^{k} e^{tx} =\sum_{n=0}^{\infty}\mathcal{B}_{n}^{(k)}(x;\theta)\frac{t^{n}}{n!}. $$

When $x=0$, we have the well-known numbers which are called Apostol–Bernoulli numbers of order *k*. That is,

$$\mathcal{B}_{n}^{(k)}(\theta)= \mathcal{B}_{n}^{(k)}(0; \theta) $$

observe that

$$\mathcal{B}_{n}(\theta)= \mathcal{B}_{n}^{(1)} (0; \theta) $$

(*cf*. [1–15]; see also the references cited therein).

Substituting $a=e$ into (1) and (2), relations between Apostol–Bernoulli numbers and polynomials are given as follows:

$$\begin{aligned} F ( t;e,q ) =& \biggl( \frac{t}{\log q}+1 \biggr) \frac{q ( q-1 ) }{qe^{t}-1}e^{tx} \\ =&\sum_{n=0}^{\infty}\mathfrak{s}_{n} ( x;e;q ) \frac{t^{n}}{n!}. \end{aligned}$$

From the above equation, we get

$$\sum_{n=0}^{\infty}\mathfrak{s}_{n} ( x;e;q ) \frac{t^{n}}{n!}=\frac{q(q-1)}{\log q}\sum_{n=0}^{\infty} \mathcal{B}_{n}(x;q)\frac{t^{n}}{n!}+\frac{q(q-1)}{t}\sum _{n=0}^{\infty}\mathcal{B}_{n}(x;q) \frac{t^{n}}{n!}. $$

Now, we equate the coefficients of $\frac{t^{n}}{n!}$ on both sides of the above equation, we deduce the following relation.

### Theorem 1

*If* $n\geq1$ *is a positive integer*, *then we obtain*

$$\mathfrak{s}_{n-1} ( x;e;q ) =\frac{q(q-1)}{\log q} \mathcal {B}_{n-1}(x;q) +\frac{q(q-1)}{n} \mathcal{B}_{n}(x;q). $$

When $q\rightarrow1$, equation (2) reduces to a generating function for Bernoulli polynomials $B_{n} (x)$. That is,

4 $$ \frac{t e^{tx}}{ e^{t} -1} =\sum_{n=0}^{\infty} \mathfrak{s}_{n} ( x;e;1 ) \frac {t^{n}}{n!}. $$

Therefore, we have

$$\sum_{n=0}^{\infty}\mathfrak{s}_{n} ( x;e;1 ) \frac{t^{n}}{n!}=\sum_{n=0}^{\infty}{B}_{n}(x) \frac{t^{n}}{n!}. $$

From the above equation, we have

$$B_{n} (x) = \mathfrak{s}_{n} ( x;e;1 ). $$

It is now clear that Bernoulli numbers $B_{n}$ are related to the numbers $\mathfrak{s}_{n} ( x;a;q )$. That is,

$$B_{n} = \mathfrak{s}_{n} ( 0;e;1 ) $$

(*cf*. [1–15], see also the references cited therein).

## New families of special numbers and polynomials of higher order

In this section, we give generating functions for new families of special numbers and polynomials of high order. By using these functions with their functional equations, we derive some formulas, identities, and relations for these numbers and polynomials.

Generating functions for new families of special numbers of order *k* are given by

5 $$\begin{aligned} F ( t;a,q;k ) =& \biggl( \frac{t\log a}{\log q}+1 \biggr)^{k} \frac{ q^{k} ( q-1 ) ^{k}}{ ( qa^{t}-1 ) ^{k}} \\ =&\sum_{n=0}^{\infty }\frak{s}_{n}^{ ( k ) } ( a;q ) \frac{t^{n}}{n!}. \end{aligned}$$

Observe that when $k=1$, we have

$$\frak{s}_{n} ( a;q ) =\frak{s}_{n}^{ ( 1 ) } ( a;q ) $$

(*cf*. [3]).

We now give a computation formula for the numbers $\frak{s}_{n}^{ ( k ) } ( a;q ) $ by the following theorem.

### Theorem 2

*Let* *k* *and* *v* *be nonnegative integers*. *Then we have*

6 $$ \frak{s}_{n}^{ ( v+k ) } ( a;q ) = \sum _{j=0}^{n} \left ( \begin{matrix} n \\ j\end{matrix} \right ) \frak{s}_{j}^{ ( v ) } ( a;q ) \frak{s}_{n-j}^{ ( k ) } ( a;q ) . $$

### Proof

We set the following functional equation:

$$F ( t;a,q;k+v ) =F ( t;a,q;v ) F ( t;a,q;k ) . $$

Combining the above equation with (4), we have

$$\sum_{n=0}^{\infty}\frak{s}_{n}^{ ( k+v ) } ( a;q ) \frac{t^{n}}{n!}=\sum_{n=0}^{\infty} \frak{s}_{n}^{ ( v ) } ( a;q ) \frac{t^{n}}{n!}\sum _{n=0}^{\infty}\frak{s}_{n}^{ ( k ) } ( a;q ) \frac{t^{n}}{n!}. $$

Therefore

$$\sum_{n=0}^{\infty}S_{n}^{ ( k+v ) } ( a;q ) \frac{t^{n}}{n!}=\sum_{n=0}^{\infty} \frac{t^{n}}{n!}\sum_{j=0}^{n}\left ( \begin{matrix} n \\ j\end{matrix} \right ) \frak{s}_{j}^{ ( v ) } ( a;q ) \frak {s}_{n-j}^{ ( k ) } ( a;q ) \frac{t^{n}}{n!}. $$

Equating the coefficients of $\frac{t^{n}}{n!}$ on both sides of the above equation, we arrive at the desired result. □

Observe that when $k=v=1$, (6) reduces to the following formula:

7 $$ \frak{s}_{n}^{ ( 2 ) } ( a;q ) =\sum _{j=0}^{n}\left ( \begin{matrix} n \\ j\end{matrix} \right ) \frak{s}_{j} ( a;q ) \frak{s}_{n-j} ( a;q ) . $$

Setting $n=0$ in (6) the above equation, we get

$$\frak{s}_{0}^{ ( 2 ) } ( a;q ) =\frak{s}_{0} ( a;q ) \frak{s}_{0} ( a;q ) =q^{2}. $$

Setting $n=1$ in (6), we get

$$\begin{aligned} \frak{s}_{1}^{ ( 2 ) } ( a;q ) =&\frak{s}_{0} ( a;q ) \frak{s}_{1} ( a;q ) +\frak {s}_{1} ( a;q ) \frak{s}_{0} ( a;q ) \\ =&2\frak {s}_{0} ( a;q ) \frak{s}_{1} ( a;q ) \\ =&\frac{2q^{2} ( q-1-q\log q ) \log a}{ ( q-1 ) \log q}. \end{aligned}$$

With the help of (4), we define the following generating function for a new family of polynomials:

8 $$\begin{aligned} G ( t,x;a,b;k ) =&a^{tx}F ( t;a,q;k ) \\ =&\sum_{n=0}^{\infty}\frak{s}_{n}^{ ( k ) } ( x;a;q ) \frac{t^{n}}{n!}. \end{aligned}$$

By combining (8) with (4), we get

$$\sum_{n=0}^{\infty}\frak{s}_{n}^{ ( k ) } ( x;a;q ) \frac{t^{n}}{n!}=\sum_{n=0}^{\infty} ( x\ln a ) ^{n}\frac{t^{n}}{n!}\sum_{n=0}^{\infty } \frak{s}_{n}^{ ( k ) } ( a;q ) \frac{t^{n}}{n!}. $$

Therefore

$$\sum_{n=0}^{\infty}\frak{s}_{n}^{ ( k ) } ( x;a;q ) \frac{t^{n}}{n!}=\sum_{n=0}^{\infty } \frac{t^{n}}{n!}\sum_{j=0}^{n}\left ( \begin{matrix} n \\ j\end{matrix} \right ) ( x\ln a ) ^{n-j}\frak{s}_{j}^{ ( k ) } ( a;q ) . $$

Equating the coefficients of $\frac{t^{n}}{n!}$ on both sides of the above equations, we get the following theorem.

### Theorem 3

*If* *n* *and* *k* *are nonnegative integers*, *then*

9 $$ \frak{s}_{n}^{ ( k ) } ( x;a;q ) =\sum _{j=0}^{n}\left ( \begin{matrix} n \\ j\end{matrix} \right ) ( x\ln a ) ^{n-j} \frak{s}_{j}^{ ( k ) } ( a;q ) . $$

By using (9), we easily see that

$$\frak{s}_{0}^{ ( k ) } ( x;a;q ) =\frak{s}_{0}^{ ( k ) } ( a;q ) . $$

Setting $k=0$ into (9), we get

$$\frak{s}_{n}^{ ( 0 ) } ( x;a;q ) =x^{n}. $$

Substituting $x=u+w$ into (8), we get

$$G ( t,u+w;a,b;k ) =a^{tu}F ( t,w;a,q;k ) $$

and

$$G ( t,u+w;a,b;k ) =a^{t ( u+w ) }F ( t,w;a,q;k ) . $$

By using the above equations, we derive the following formulas, respectively:

10 $$ \frak{s}_{n}^{ ( k ) } ( u+w;a;q ) =\sum _{j=0}^{n}\left ( \begin{matrix} n \\ j\end{matrix} \right ) ( u\ln a ) ^{n-j} \frak{s}_{j}^{ ( k ) } ( w;a;q ) $$

and

11 $$ \frak{s}_{n}^{ ( k ) } ( u+w;a;q ) =\sum _{j=0}^{n}\left ( \begin{matrix} n \\ j\end{matrix} \right ) \bigl( ( u+w ) \ln a \bigr) ^{n-j}\frak{s}_{j}^{ ( k ) } ( a;q ) . $$

Combining (10) with (11), we get the following theorem.

### Theorem 4

*If* *n* *is a nonnegative integer*, *then*

12 $$\begin{aligned}& \sum_{j=0}^{n}\left ( \begin{matrix} n \\ j\end{matrix} \right ) ( \ln a )^{n-j}\frak{s}_{j}^{ ( k ) } ( a;q ) \sum _{m=0}^{n-j}\left ( \begin{matrix} n-j \\ m\end{matrix} \right ) u^{m}w^{n-j-m} \\& \quad =\sum_{j=0}^{n}\left ( \begin{matrix} n \\ j\end{matrix} \right ) ( u\ln a )^{n-j}\frak{s}_{j}^{ ( k ) } ( w;a;q ) . \end{aligned}$$

Substituting $w=1$ into (12), we get

$$\begin{aligned}& \sum_{j=0}^{n}\left ( \begin{matrix} n \\ j\end{matrix} \right ) ( \ln a ) ^{n-j}\frak{s}_{j}^{ ( k ) } ( a;q ) \sum _{m=0}^{n-j}\left ( \begin{matrix}n-j \\ m\end{matrix} \right ) u^{m} \\& \quad =\sum_{j=0}^{n}\left ( \begin{matrix} n \\ j\end{matrix} \right ) ( u\ln a ) ^{n-j}\frak{s}_{j}^{ ( k ) } ( 1;a;q ) \\& \quad =\frak{s}_{n}^{ ( k ) } ( u+1;a;q ) . \end{aligned}$$

Thus we also get the following results:

$$\frak{s}_{n}^{ ( k ) } ( u-1;a;q ) =\sum _{j=0}^{n}\left ( \begin{matrix} n \\ j\end{matrix} \right ) ( u-1 ) ^{n-j} ( \ln a ) ^{n-j}\frak {s}_{j}^{ ( k ) } ( a;q ) $$

and

$$\frak{s}_{n}^{ ( k ) } ( u+1;a;q ) =\sum _{j=0}^{n}\left ( \begin{matrix} n \\ j\end{matrix} \right ) ( u+1 )^{n-j} ( \ln a ) ^{n-j}\frak {s}_{j}^{ ( k ) } ( a;q ) . $$

From the above equations, we obtain the following corollary.

### Corollary 5

*If* *n* *is a nonnegative integer*, *then*

$$\frak{s}_{n}^{ ( k ) } ( u-1;a;q ) +\frak {s}_{n}^{ ( k ) } ( u+1;a;q ) =n!\sum_{j=0}^{n} \sum _{k=0}^{n-j} \frac{ 1+(-1)^{n-j-k}}{j! k!(n-j-k)!} u^{k} ( \ln a ) ^{n-j}\frak{s}_{j}^{ ( k ) } ( a;q ) . $$

## Recurrence relations and derivative formulas

In this section, by using partial derivative formulas for generating functions of new families of special numbers and polynomials of higher order, we derive recurrence relations for these numbers and polynomials.

### Theorem 6

*If* *n* *is a nonnegative integer*, *then*

$$\frak{s}_{n}^{ ( k ) } ( x;a;q ) =\frac{\partial }{\partial x} \bigl\{ G(t,x;a,b;k) \bigr\} =n \log a \frak{s}_{n-1}^{ ( k ) } ( x;a;q ) . $$

### Proof

Taking partial derivative of Equation (8) with respect to *x*, we get the following derivative formula:

$$\frac{\partial}{\partial x} \bigl\{ G(t,x;a,b;k) \bigr\} =t \log a G(t,x;a,b;k) . $$

By combining the above equation with (8), we obtain

$$\sum_{n=0}^{\infty}\frak{s}_{n}^{ ( k ) } ( x;a;q ) \frac{t^{n}}{n!} = \log a \sum_{n=0}^{\infty}\frak{s}_{n}^{ ( k ) } ( x;a;q ) \frac{t^{n+1}}{n!}. $$

Making some elementary calculations in the above equation after equating the coefficients of $\frac{t^{n}}{n!}$ on both sides of the final equation, we arrive at the desired result. □

### Theorem 7

(Recurrence relation)

*If* *n* *is a nonnegative integer*, *then*

13 $$\begin{aligned} \frak{s}_{n+1}^{ ( k ) } ( a;q ) =& \biggl( k- \frac{n}{\log q} \biggr) \log a \frak{s}_{n}^{ ( k ) } ( a;q ) \\ &{}+\frac{kq}{q(q-1)} \sum_{j=0}^{n} \left ( \begin{matrix} n \\ j\end{matrix} \right ) (\log a )^{n+1-j} \frak{s}_{j}^{ ( k+1 ) } ( a;q ). \end{aligned}$$

### Proof

Taking partial derivative of Equation (4) with respect to *t*, we get the following derivative formula:

$$\biggl(\frac{t\log a}{ \log q} +1 \biggr) \frac{\partial}{ \partial t } \bigl\{ F(t;a,q;k)\bigr\} =k \log a F(t;a,q;k) +\frac{kq \log a}{q(q-1)} F(t;a,q;k+1). $$

By combining the above equation with Equation (4), we get

$$\begin{aligned}& \biggl(\frac{t\log a}{ \log q} +1 \biggr) \sum_{n=1}^{\infty} \frak{s}_{j}^{ ( k ) } ( a;q ) \frac{t^{n-1}}{(n-1)!} \\& \quad =k \log a \sum_{n=0}^{\infty} \frak{s}_{n}^{ ( k ) } ( a;q ) \frac{t^{n}}{n !} + \frac{kq \log a}{q(q-1)} \sum_{n=0}^{\infty} (\log a)^{n} \frac {t^{n}}{n !} \sum_{n=0}^{\infty} \frak{s}_{n}^{ ( k ) } ( a;q ) \frac{t^{n}}{n !}. \end{aligned}$$

By using the Cauchy product on the right-hand side of the above equation, we get

$$\begin{aligned}& \biggl(\frac{t\log a}{ \log q} +1 \biggr) \sum_{n=1}^{\infty} \frak{s}_{j}^{ ( k ) } ( a;q ) \frac{t^{n-1}}{(n-1)!} \\& \quad =k \log a \sum_{n=0}^{\infty} \frak{s}_{n}^{ ( k ) } ( a;q ) \frac{t^{n}}{n !} + \frac{kq \log a}{q(q-1)} \sum_{n=0}^{\infty} \sum _{j=0}^{n} \left ( \begin{matrix} n \\ j\end{matrix} \right ) (\log a )^{n-j} \frak{s}_{j}^{ ( k+1 ) } ( a;q ) \frac{t^{n}}{n !}. \end{aligned}$$

Making some elementary calculations in the above equation, after that equating coefficients of $\frac{t^{n}}{n !}$ on both sides of the final equation, we arrive at the desired result. □

Replacing *a* by *e* in (13), we get the following corollary.

### Corollary 8

*If* *n* *is a nonnegative integer*, *then*

$$\frak{s}_{n+1}^{ ( k ) } ( e;q ) =\biggl(k-\frac{n}{\log q} \biggr)\frak{s}_{n}^{ ( k ) } ( e;q ) + \frac{kq}{q(q-1)} \sum _{j=0}^{n}\left ( \begin{matrix} n \\ j\end{matrix} \right ) \frak{s}_{j}^{ ( k+1 ) } ( e;q ) . $$

### Theorem 9

*If* *n* *and* *k* *are nonnegative integers*, *then*

$$\begin{aligned} \frak{s}_{n}^{ ( k ) } ( e;q ) =& q^{k} (q-1)^{k} \sum_{j=0}^{k}\left ( \begin{matrix} k \\ j \end{matrix} \right ) \frac{1}{(\log q)^{j}} \frac{1}{(k-1)^{j}} \\ &{} \times \sum_{m=0}^{n}\left ( \begin{matrix} n \\ m \end{matrix} \right ) \left ( \begin{matrix} n+k-j \\ k-j\end{matrix} \right )^{-1} \mathcal{B}_{n}^{(j)}(q ) \mathcal{B}_{n-m}^{(k-j)}(q ) . \end{aligned}$$

### Proof

We set the following functional equation:

$$F(t;e,q;k) = \sum_{j=0}^{k}\left ( \begin{matrix} k \\ j \end{matrix} \right ) \frac{ q^{k} (q-1)^{k} t^{j-k}}{(\log q)^{j}} F_{AB} (t,0;q;j) F_{AB} (t,0;q;;k-j). $$

Combining the above equation with (3) and (4), we get

$$\sum_{n=0}^{\infty} \frak{s}_{n}^{ ( k ) } ( e;q ) \frac{t^{n}}{n!} =\sum_{j=0}^{k} \left ( \begin{matrix} k \\ j \end{matrix} \right ) \frac{ q^{k} (q-1)^{k} t^{j-k}}{(\log q)^{j}} \sum_{n=0}^{\infty} \mathcal{B}_{n}^{(j)}(q ) \frac{t^{n}}{n!} \sum _{n=0}^{\infty}\mathcal{B}_{n}^{(k-j)}(q ) \frac{t^{n}}{n!}. $$

Therefore

$$\sum_{n=0}^{\infty} \frak{s}_{n}^{ ( k ) } ( e;q ) \frac{t^{n}}{n!} =\sum_{j=0}^{k} \left ( \begin{matrix} k \\ j \end{matrix} \right ) \frac{ q^{k} (q-1)^{k} }{(\log q)^{j}} \sum_{n=0}^{\infty}\sum _{m=0}^{n} \left ( \begin{matrix} n \\ m \end{matrix} \right ) \mathcal{B}_{m}^{(j)}(q ) \mathcal{B}_{n-m}^{(k-j)}(q ) \frac{t^{n+j-k}}{n!}. $$

Making some elementary calculations in the above equation, after that equating the coefficients of $\frac{t^{n}}{n!}$ on both sides of the final equation, we arrive at the desired result. □

## Conclusion

Recently, many researchers have investigated and studied new families of special numbers and polynomials since these numbers and polynomials are used not only in science, but also in social sciences. That is, these numbers and polynomials have many applications in mathematics, in probability and statistics, in physics, in engineering, and in economic problems. Especially, polynomials have basic operations, which are addition, subtraction, multiplication, polynomials. Therefore, researchers can use them. In work of Simsek and Yardimci [12], we see that polynomials are used as models, related to differential equations, for real word problems related to sciences, approximate or curve fit experimental data, calculate beam deflection under loading, represent some properties of gases, and perform computer-aided geometric design in engineering. Polynomials represent the characteristics of a linear dynamic system, and we also know that a ratio of two polynomials represents a transfer function of a linear dynamic system. With the help of polynomials, one defines basis used in finite element computation and constructs parametric curves. Due to applications of the special numbers and polynomials with their generating functions, in this paper we investigate and study new families of special numbers and polynomials of higher order. We give their generating functions including their functional equations and partial derivative formulas.

By using these functions, we give many new identities, derivative formulas, and recurrence relations for these new families of special numbers and polynomials of higher order. These identities and relations will be potentially used in mathematics, in probability and statistics, in physics, in engineering, and in economic problems.
